# Incubation of Infants

**Published:** 1883-07

**Authors:** 


### INCUBATION OF INFANTS.

The *Paris Temps* asserts that Dr. Tavernier has discovered means of extending his system of artificial incubation indefinitely, and is successfully applying it to infants born at a comparatively early period of gestation.

